# Radiation-Induced Graft Immobilization (RIGI): Covalent Binding of Non-Vinyl Compounds on Polymer Membranes

**DOI:** 10.3390/polym13111849

**Published:** 2021-06-02

**Authors:** Martin Schmidt, Stefan Zahn, Florian Gehlhaar, Andrea Prager, Jan Griebel, Axel Kahnt, Wolfgang Knolle, Robert Konieczny, Roger Gläser, Agnes Schulze

**Affiliations:** 1 Leibniz Institute of Surface Engineering (IOM), Permoserstr. 15, 04318 Leipzig, Germany; martin.schmidt@iom-leipzig.de (M.S.); stefan.zahn@iom-leipzig.de (S.Z.); florian.gehlhaar@studserv.uni-leipzig.de (F.G.); andrea.prager@iom-leipzig.de (A.P.); jan.griebel@iom-leipzig.de (J.G.); axel.kahnt@iom-leipzig.de (A.K.); wolfgang.knolle@iom-leipzig.de (W.K.); robert.konieczny@iom-leipzig.de (R.K.); 2 Institute of Chemical Technology, Leipzig University, Linnéstraße 3, 04103 Leipzig, Germany; roger.glaeser@uni-leipzig.de

**Keywords:** polymer surface, membrane, radiation-induced grafting, electron beam, reaction mechanism, DFT, MD

## Abstract

Radiation-induced graft immobilization (RIGI) is a novel method for the covalent binding of substances on polymeric materials without the use of additional chemicals. In contrast to the well-known radiation-induced graft polymerization (RIGP), RIGI can use non-vinyl compounds such as small and large functional molecules, hydrophilic polymers, or even enzymes. In a one-step electron-beam-based process, immobilization can be performed in a clean, fast, and continuous operation mode, as required for industrial applications. This study proposes a reaction mechanism using polyvinylidene fluoride (PVDF) and two small model molecules, glycine and taurine, in aqueous solution. Covalent coupling of single molecules is achieved by radical recombination and alkene addition reactions, with water radiolysis playing a crucial role in the formation of reactive solute species. Hydroxyl radicals contribute mainly to the immobilization, while solvated electrons and hydrogen radicals play a minor role. Release of fluoride is mainly induced by direct ionization of the polymer and supported by water. Hydrophobic chains attached to cations appear to enhance the covalent attachment of solutes to the polymer surface. Computational work is complemented by experimental studies, including X-ray photoelectron spectroscopy (XPS) and fluoride high-performance ion chromatography (HPIC).

## 1. Introduction

Over the past few decades, membrane technology has gradually developed into a popular separation technology that is widely used in various fields such as water treatment [1], clinical medicine [2], or food processing [3]. The use of membranes for industrial processes has many significant advantages such as reduced energy consumption, gentle product treatment, and modularity and scalability [4]. The global market for membrane modules was estimated to be worth 12.4 billion USD in 2008, with polymer-based membranes accounting for 92.1% [5]. Polyether sulfone (PES) and polyvinylidene fluoride (PVDF) predominate, with an increasing tendency for PVDF in industrial applications [6]. For this reason, polyvinylidene fluoride has received much attention in recent years [7,8]. PVDF is a semi-crystalline polymer with the structural unit –(CH_2_-CF_2_)_n_– and numerous exceptional properties such as high mechanical strength, thermal stability, chemical resistance (especially against acids and oxidizing agents [9]), and good processability [7]. A major disadvantage of such fluorocarbon polymers is their strong hydrophobicity, since most applications (e.g., water treatment or biomedicine) require hydrophilic polymer surfaces [7]. In addition, surface modification is necessary to create functional groups so that smart and tailored materials can be produced [8]. So far, only few approaches have been applied for this purpose, including physical coating and chemical grafting [7].

### 1.1. Polymer Surface Grafting

**Figure 1:**
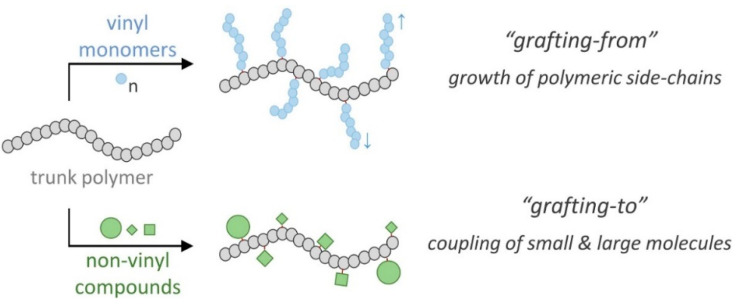
Comparison of the two most common grafting approaches: grafting from and grafting to.

According to the IUPAC, polymer surface grafting is characterized by the “generation of active sites that can lead to the initiation of a graft polymerization” [10], which consequently implies the formation of graft copolymers. Some authors [11] provide a broader definition of grafting as covalent binding of general modifying compounds on the trunk polymer, thus including not only graft copolymers but also small and large single molecules as a result of novel research in recent years [12,13]. Grafting is a promising method for polymer surface modification, as long-term chemical stability through covalent bonds avoids delamination [14]. Two main approaches can be distinguished: (a) the grafting-from approach, in which polymeric side chains grow directly from the surface by polymerizing (mostly vinyl) monomers, and (b) the grafting-to approach, in which prefabricated species such as polymers or single molecules are attached to the surface (Figure 1) [11,15]. A variety of grafting techniques have been investigated [16,17,18], and free-radical techniques are the oldest and most widely used methods [19]. The initiation of free-radical reactions can be achieved by various means such as chemical initiators (e.g., AIBN [20]), redox reactions [21], UV light [22], plasma treatment [23], or high-energy ionizing radiation (radiation-induced grafting, RIG [24]).

### 1.2. Radiation-Induced Grafting (RIG)

**Figure 2:**
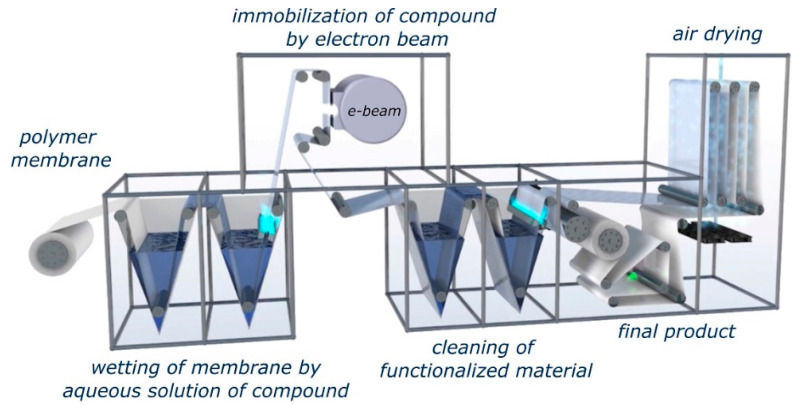
Illustration of our platform technology for roll-to-roll processing of polymer membranes at the Leibniz Institute of Surface Engineering (IOM).

Polymer functionalization via grafting requires the activation of the trunk polymer, which can be achieved by an irradiation step with high-energy (i.e., ionizing) radiation, including γ-rays, X-rays, or electron beams (EBs) [25]. Ionizing radiation interacts with matter through the electrons of the atomic shell, resulting in ionization or excitation and, thus, in reactive species (usually free radicals) [26]. These species can serve as starting points for chemical reactions such as polymerization or radical recombination. Radiation-induced grafting (RIG) offers advantages such as simplicity, control over reaction parameters and graft content, and the ability to use commercially available polymer substrates [27]. In addition, RIG methods are usually unparalleled clean and environmentally friendly as no potentially harmful substances such as catalysts, additives, initiators, or organic solvents are required, providing a tremendous benefit for biomedical products [12,16]. Besides the peroxide method, two types of implementation are of particular interest for technical applications [28]: (1) pre-irradiation method, where only the trunk polymer is irradiated at an irradiation facility and returned cooled (resulting in metastable radicals), followed by grafting with the desired monomer in a separate step, and (2) simultaneous method, where the trunk polymer is impregnated with a solution of the grafting compound and irradiated simultaneously in one step, which is preferred in terms of continuous operation, especially when using electron beams [29]. Compared to γ-rays, EB irradiation offers significant advantages for potential industrial applications, such as fast processing, variable penetration depth, no radiation hazard, ease of operation, and an environmentally friendly process (Figure 2) [30,31,32].

### 1.3. RIG: The Grafting-from Approach

**Figure 3:**
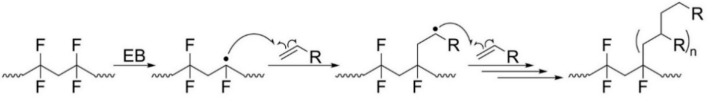
Conventional vinyl polymerization mechanism proposed in RIGP.

In the literature, radiation-induced grafting is almost exclusively referred to and performed as radiation-induced graft polymerization (RIGP), with initial work dating back to 1960 [33,34]. Grafting-from strategies such as RIGP are based on a radical-mediated vinyl polymerization mechanism (Figure 3) [35]. Important applications of RIGP can be found in many fields [27,32,36,37,38,39,40,41]. Advantages include a high density of functional groups due to side-chain formation [42]. Disadvantages include the restriction to polymerizable monomers, mostly vinyl compounds such as acrylates, allyls, and styrenics [27]. Many of these substances are known to have environmental [43] and aquatic toxicity [44], cytotoxicity [45], and allergenic properties [46], requiring additional purification steps to remove the unreacted monomer.

### 1.4. RIG: The Grafting-to Approach

In recent years, a new RIG method has been introduced that complies with the grafting-to approach. Briefly, non-vinyl grafting compounds (i.e., molecules that do not contain a reactive polymerizable group, usually a vinyl group) can be covalently immobilized on polymer membranes in a one-step EB irradiation process [11,12]. Initial work was published by Schulze et al. in 2010 [12], immobilizing small organic molecules, such as malonic acid, *p*-toluenesulfonic acid, triethylamine, glycerol, taurine, and others, on PES membranes. Later, these small molecules were successfully coupled to different types of polymer membranes [13]. In the grafting-to approach, not only small molecules but also other prefabricated species such as synthetic hydrophilic polymers, e.g., polyethylene glycol (PEG), can be covalently coupled [47]. Furthermore, biological macromolecules such as proteins/enzymes [48] are successfully immobilized on membranes using this electron-beam-based procedure. Thus, additional chemical coupling steps such as EDC/NHS [49] might be suspended. Further specialized compounds with different functionalities have been grafted on membranes, e.g., photosensitizers [50], antimicrobial peptides [51], or piperazine, in order to produce hydrophilic amide layers [52]. However, there are scarcely any publications dealing with this novel type of grafting mechanism, since RIG is typically performed exclusively with vinyl monomers (i.e., RIGP). So far, a similar application was found involving the use of the non-vinyl carbon tetrabromide CBr_4_ to introduce bromide functionalities for subsequent ATRP-controlled methacrylate grafting [53].

**Figure 4:**
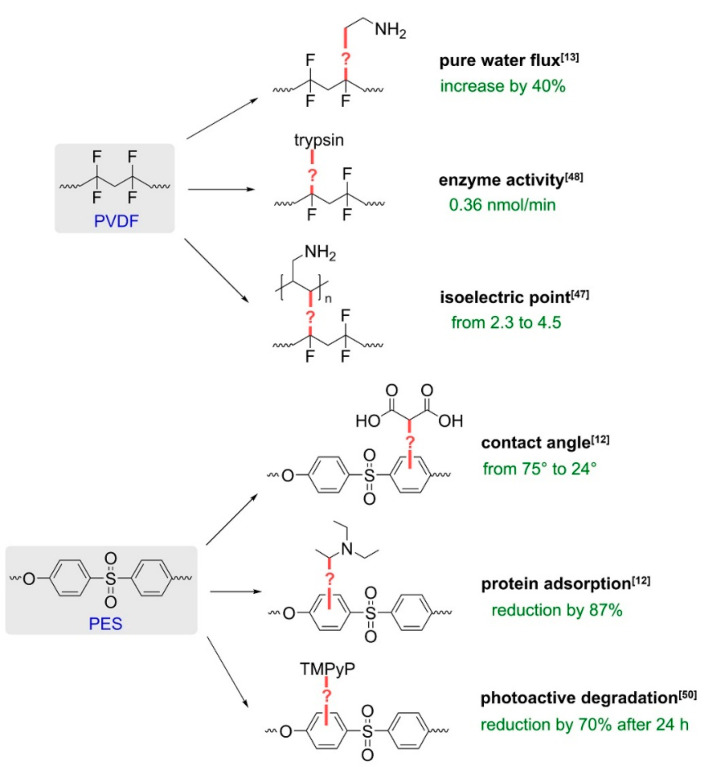
Selection of successful membrane modifications via the RIGI approach. Despite significant improvements, the way of binding the modifying compounds is still unclear.

However, CBr_4_ is a reactive compound and hence not comparable with the covalent immobilization of stable organic molecules such as glycerol [12] or PEG [47]. In addition, imprecise terminology impedes a clear overview. In earlier publications, terms such as “radiation immobilization” [54] or “electron beam immobilization” [55] were used; however, they actually refer to radiation-induced polymerization of a hydrogel [56], although it was already suspected that “radiation itself may lead to chemical bonding between the biomaterials and the polymer matrix” [40]. Therefore, we propose a new term, radiation-induced graft immobilization (RIGI), in analogy to the well-known RIGP, emphasizing the direct and covalent attachment of non-reactive compounds. Despite a considerable number of published studies demonstrating the significant improvements of polymer materials modified via RIGI, the actual binding of the modifying compounds used is still unclear (Figure 4).

### 1.5. Aims of This Study

So far, there is no published in-depth reaction mechanism for the RIGI approach (Figure 4), whereas the mechanism of RIGP (Figure 3) is generally accepted and intensively investigated. In this study, we proposed a reaction mechanism for the EB-induced immobilization of non-vinyl substances on polymeric materials in order to provide a scientific foundation for this promising new method. We used two small model molecules, glycine and taurine, to investigate the covalent coupling on PVDF membranes by computational as well as experimental studies. Kohn–Sham density functional theory (DFT) calculations and tight-binding-based molecular dynamics (MD) simulations were carried out to understand the mode of action. The experimental challenges in this study were the mono-molecular binding of modifying agents and the poor solubility of PVDF, resulting in a limited experimental and analytical repertoire. By using the amino acid glycine, a suitable bridge for the immobilization of proteins/enzymes is provided, which may be one of the most attractive future applications of RIGI and part of current research.

## 2. Materials and Methods

### 2.1. Computational Methods

Investigated structures were optimized on a DFT level using the TURBOMOLE suite [57] for which the BLYP functional [58] in combination with a TZVP basis set [59] was employed. The SCF convergence criterion was increased to 10^−8^ Hartree, and the resolution of identity approximation [60] accelerated the calculations. Additionally, the third version of Grimme’s empirical dispersion correction [61] in combination with Becke–Johnson damping improved the description of weak non-covalent interactions. Water solvation was considered by the COSMO model [62] (relative dielectric constant 80.4, refractive index 1.33). Minima and transition states were confirmed by seminumerical frequency calculations. Thermodynamic corrections were calculated with freeh, a tool provided by TURBOMOLE. Values were corrected by the electronic entropy *S_el_*, which is given by

(1) $$S_{el}=R\cdot \ln M_{s}$$

where *R* is the universal gas constant, while *M_S_* is the multiplicity of the compound. The electronic energy was obtained from single-point calculations with a revised version of B3LYP [63]. As shown by Reiher, B3LYP* reduces significantly the error for the energy gap between different spin states. We selected this functional to reduce errors from radical recombination reactions (two doublet spin states forming a singlet spin state) and the reaction of a solvated electron with the compounds (a singlet spin state is changed to a doublet spin state since the energy of the electron is taken from the literature) due to the change in spin. The single-point calculations were carried out with ORCA [64], where CPCM [65] was selected as the solvation model of water. Furthermore, the RIJCOSX approach was employed to speed up the calculation of exchange and Coulomb integrals [66]. Again, the empirical dispersion correction of Grimme and a TZVP basis were selected for the electronic structure calculations. Gibbs free energy of the solvated electron (−156.6 kJ/mol) was taken from the literature [67]. All input and output files of the static quantum chemistry calculations were uploaded to the NOMAD repository and are available using the following PID: HpfuIDx9Sfqfza0CFEOmFQ.

We employed in our computational studies a small model chain for PVDF, 1,1,3,3,5,5,7,7-octafluorooctan (**1a**). Convergence of the obtained reaction energies with the chain length were validated in two examples: the abstraction of a hydrogen and of a fluorine atom. We observed a convergence of the obtained reaction energy within 4 kJ/mol already for a model consisting of 3 monomer units.

In addition to the static quantum chemistry calculations, the reaction space of obtained intermediates was screened by tight-binding-based molecular dynamics simulations for which the xtb-program version 6.3.2 was employed [68,69]. Each molecular compound was centered in a water solvation sphere with a diameter between 14 and 18 Å. This was achieved by a space-fixed origin with a time-independent wall potential. Initial coordinates of the water molecules were obtained from a classical MD simulation employing the TIP3P model, where water molecules were removed if an atom was closer to the solute than 2 Å. A second wall potential between 8 and 11 Å was added for the atoms of the solute to keep them within the water shell. Each system was equilibrated by a NVT simulation, where the temperature was kept constant by a Berendsen thermostat [70]. This includes an initial step of about 10 ps with constrained bonds and subsequently a run of at least 10 ps without any restriction to the bond topology. If the solute stayed stable, a metadynamics simulation was added, where a potential based on standard root-mean-square deviation (RMSD) in Cartesian space was employed to screen the reaction space. This potential was restricted to heavy atoms and hydrogen atoms bonded to carbon atoms of the solute (scaling factor of RMSD criteria 0.2; width of Gaussian potential 1.2). To identify reactions induced by the solvated electron, we carried out at least 50 MD runs without a bias potential of 10 ps run time.

### 2.2. Chemicals and Materials

A commercially available polyvinylidene fluoride flat sheet microfiltration membrane (PVDF, ROTI^®^, 0.45 µm) was purchased from Carl Roth (Karlsruhe, Germany). In addition, the following chemicals were used: NaOH (1 M), perchloric acid (70%), and buffer solution pH 10 (TITRINORM^®^) from VWR (Radnor, PA, USA); *tert*-butyl alcohol (tBuOH, 99.5%) from Alfa Aesar (Karlsruhe, Germany); and nitrous oxide (N_2_O, 99.95%) from Air Liquide (Düsseldorf, Germany). Glycine, taurine, ethyl sodium sulfate (C_2_SS), dodecyl sodium sulfate (C_12_SS, SDS), tetramethylammonium bromide (C_1_TAB), and octyltrimethylammonium bromide (C_8_TAB) were obtained from Sigma-Aldrich (St. Louis, MO, USA). Furthermore, 2-aminoethyl methacrylate hydrochloride (AEMA, 90%, stabilized) from Acros Organics (Geel, Belgium) was used. Deionized water of Millipore^®^ quality and EtOH (absolute; Merck, Darmstadt, Germany) were used for all steps. All chemicals were of analytical grade and used without further purification.

### 2.3. Electron Beam Irradiation of Pristine PVDF

Irradiation studies with PVDF were carried out to investigate the release of hydrogen fluoride (HF) under different conditions in order to obtain insights into possible reaction paths. Due to the volatile nature of hydrogen fluoride, PVDF samples (Ø = 33 mm, m = 63.4 mg ± 0.4 mg) were irradiated in 10 mL glass flasks sealed with septa. For complete penetration of the glass flask, a high-energy 10 MeV linear electron accelerator (MB10-30MP; Mevex, Stittville, ON, Canada) was used. The dosage of irradiation was varied between 0 and 150 kGy. PVDF samples were irradiated either dry or wet (i.e., pristine or impregnated with aqueous solutions, respectively), however, always under N_2_ or N_2_O atmosphere to minimize hydroperoxide formation in presence of O_2_. During electron beam treatment, the samples were cooled to prevent temperature-induced degradation. All experiments were performed at least in triplicate.

The influence of water during irradiation was examined by using wet membranes. Due to the hydrophobic nature of PVDF, a pre-wetting step had to be applied (0.5 min EtOH abs., 5 × 5 min H_2_O in excess to exchange EtOH). The water-wetted membrane samples were partially dried by placing them in a laboratory tissue and applying a roller (m = 330 g). Wet membranes were transferred quickly to glass flasks, sealed with septa, and purged with N_2_ (100 mL/min, 20 s). This method allowed reproducible water contents at each irradiation (mass fraction of water ω = 43.3% ± 1.1%). Higher water amounts were achieved by skipping the drying step (ω = 57.9% ± 1.3%). To study the effect of water radiolysis products, PVDF membranes were impregnated with aqueous solutions of radical scavengers instead of pure water (perchloric acid solution at pH < 3, NaOH solution at pH 11, saturated N_2_O solution, tBuOH with volume fraction of φ = 2.5%, or a combination of the latter). In the case of N_2_O as scavenger, the flasks were also purged with N_2_O (100 mL/min, 20 s). Directly after irradiation, 10.0 mL of buffer (pH 10) was injected into the sealed flask using a syringe in order to extract released HF via dissociation. The extraction was performed for 1 h by shaking at 450 rpm. Subsequently, aliquots were taken for the quantification of the fluoride content using high-performance ion chromatography (HPIC), as well as a commercially available photometric fluoride assay (Spectroquant^®^; Merck, Darmstadt, Germany), adapted to 48-well microplates in a microplate reader (Infinite^®^ M200 Pro; Tecan, Groedig, Austria).

Fluoride analysis was performed on a Dionex Integrion HPIC system equipped with a DS6 heated conductivity cell. The Chromeleon 7 Chromatography Data System was used for instrument control, data collection, and processing (Thermo Fisher Scientific, Sunnyvale, CA, USA). For automated online sample preparation, an InGuard Na/HRP (9 mm × 24 mm) column was used for cleanup, and an IonPac UTAC-LP2 (4 mm × 35 mm) column was connected as ultratrace anion concentrator. The analysis of the target analyte anion was performed using the Dionex IonPac AS19 Anion-Exchange Column (2 mm × 250 mm, 4 μm), with a Dionex IonPac AS19 Guard Column (2 mm × 50 mm, 4 µm) coupled to an anion self-regenerating Dionex ASRS 500 suppressor (Thermo Fisher Scientific, Sunnyvale, CA, USA). A Dionex AS-DV autosampler (Thermo Fisher Scientific, Sunnyvale, CA, USA) was used for sample processing. A sample volume of 4 µL was injected. The eluent was generated using an EGC-500 KOH eluent generator cartridge. The operating parameters were as follows: a temperature of 30 °C, a flow rate of 0.25 mL min^−1^, suppressor constantly at 28 mA, and eluent: isocratic 10 mM (10 min), linear until 45 mM (15 min), isocratic 45 mM (2 min), and back to 10 mM for equilibration until end of run time after 34 min.

### 2.4. Electron Beam Grafting

Grafting according to the RIGI principle was studied by using two small organic molecules, glycine and taurine, applying the following procedure: First, the hydrophobic PVDF membrane had to be pre-wetted, as described earlier. Subsequently, membrane samples (Ø = 47 mm) were impregnated for 10 min with 5 mL of an aqueous solution of glycine or taurine, each with a mass fraction of ω = 0.01%, ω = 0.1%, or ω = 1%. Impregnated membranes were placed on a glass plate without further drying and irradiated by electron beam in an N_2_ atmosphere with O_2_ quantities <15 ppm using a self-built low-energy electron accelerator. The voltage and conveyor speed were 160 kV and 2.8 m/min, respectively. An irradiation dosage of 0 kGy (reference), 50 kGy, or 150 kGy was applied by adjusting the beam current. Directly after EB irradiation, a washing step at 450 rpm was performed to remove non-covalently attached substances (5 min H_2_O, 0.5 min EtOH abs., 5 × 20 min H_2_O in excess). Finally, the modified membrane samples were dried at 37 °C overnight and used for further characterization.

In addition to the non-vinyl RIGI substrates (glycine and taurine), immobilization of AEMA as an RIGP vinyl monomer was performed in order to compare grafting yields. Aqueous AEMA solutions were used (ω = 2% and ω = 0.2%, corresponding to c = 123 mM and c = 12 mM, respectively, which was comparable to the glycine impregnation solutions of ω = 1% and ω = 0.1%, corresponding to c = 135 mM and c = 13 mM, respectively). To study the influence of water on grafting yield, combinations of simultaneous and pre-irradiation methods were examined (glycine, ω = 0.1%, 150 kGy). The simultaneous method was performed with wet-impregnated membranes corresponding to the general procedure given above or with impregnated but dried membranes (same procedure, but samples were dried overnight at 50 °C prior to irradiation in order to remove water). Pre-irradiation methods were performed by previous irradiation of only water-wetted membranes or dry membranes. Directly after irradiation, the impregnation step was carried out as in the general procedure. The effect of hydrophobicity, and of charge, on the grafting yield was studied by using 10 mM of soluble surfactants with different chain lengths each (cationic C_n_-trimethylammonium bromide (C_n_TAB) with C_1_TAB and C_8_TAB, as well as anionic alkyl sodium sulfate (C_n_SS) with ethyl sodium sulfate (C_2_SS) and dodecyl sodium sulfate (C_12_SS)).

The chemical composition of pristine and modified PVDF membranes was investigated by X-ray photoelectron spectroscopy (XPS; AXIS Ultra; Kratos Analytical, Manchester, UK) with a monochromatic Al K_α_ source operated at 150 W (15 kV and 10 mA). Charge compensation and correction were applied to record survey spectra (160 eV pass energy, 1.0 eV resolution) and high-resolution spectra (40 eV pass energy, 0.1 eV resolution), respectively.

## 3. Results and Discussion

### 3.1. Reactivity of PVDF

#### 3.1.1. Reactions Induced by Products of Water Radiolysis

The initial degree of ionization by the electron beam is proportional to the amount of electrons in the whole system. Thus, water radiolysis should play an essential role for the immobilization of molecular compounds. The products of water radiolysis are well known and result in the formation of solvated electrons, OH radicals, and H radicals as reactive intermediates [71]. Additionally, compounds with low reactivity, such as H_2_ and H_2_O_2_, are formed due to in-spur recombination. At pH 7, the water radiolysis was estimated to yield about 4.8 hydroxyl radicals per 100 eV energy of the electron beam after 10 fs. Furthermore, 4.8 solvated electrons are formed after 1 ps. However, follow-up reactions reduce the number of hydroxyl radicals and solvated electrons while new reactive species are formed. These are hydrogen radicals and hydrogen peroxide. After the in-spur recombination processes have taken place, around 2.7 solvated electrons per 100 eV energy absorbed from ionizing radiation, ~2.6 OH radicals per 100 eV, and approximately 0.5 H radicals per 100 eV are generated [71]. Hydrogen peroxide is not expected to react with PVDF. Thus, solvated electrons and hydroxyl radicals are considered to be most important for RIGI, while hydrogen radicals likely play a minor role due to their lower concentration. To understand the role of each reactant, we carried out computational and experimental studies.

**Scheme 1:**
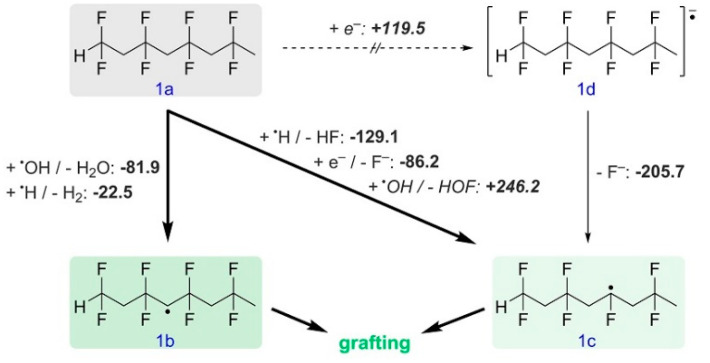
Investigated reactions of PVDF with radiolysis products of water. Most important for RIGI is **1b** and to a lesser extent **1c**. All free reaction enthalpies are given in kJ/mol.

For surface grafting, the region of interest is the polymer–water interface. Scheme 1 shows the computational results (free reaction enthalpies) for reactions of PVDF (**1a**) with products of water radiolysis. The OH radical can solely abstract a hydrogen atom of PVDF, forming **1b**. The reaction to **1c** is strongly endergonic and, thus, can be excluded. In contrast, the hydrogen radical can abstract a H as well as a F atom. All these reactions proceed without a significant reaction barrier. In the case of the solvated electron, a reaction with PVDF appears questionable since the reaction from **1a** to **1d** is strongly endergonic. The C-F bond splitting in **1a**, forming F^−^ and **1c**, seems possible since it is exergonic. However, our approach does not allow determining reaction barriers for this reaction. Thus, hydroxyl radicals and **1b** are most important for RIGI at PVDF, while **1c** is likely play a minor role due to the low concentration of hydrogen radicals in solution.

#### 3.1.2. Direct Ionization of PVDF

**Scheme 2:**
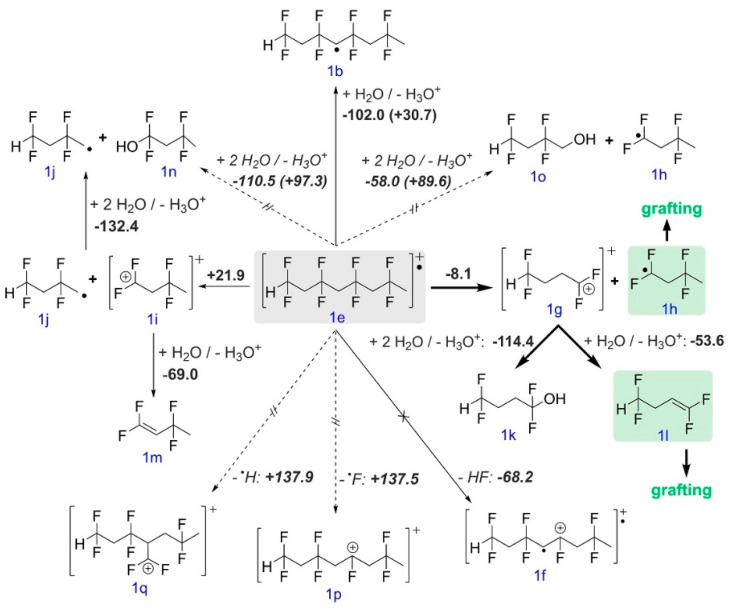
Reactions of cationic PVDF in the ground state, formed due to electron beam irradiation, in the presence of water. Most important for RIGI are **1h** and **1l**. Free reaction enthalpies are given in kJ/mol. Values in brackets are energy barriers of transition states. Please note, the reaction from **1e** to **1f** is exergonic, but a direct reaction pathway for intramolecular dehydrofluorination could not be found.

Scheme 2 shows the reactions of ionized PVDF **1e**, formed due to irradiation with high-energy electrons, in the presence of water. Abstraction of a hydrogen atom by water from **1e**, forming **1b** and a hydronium ion, is exergonic and possesses a significant activation energy of 30.7 kJ/mol. The release of HF (dehydrofluorination), resulting in **1f**, is not possible since a direct reaction path could be excluded by scanning the reaction coordinate. A chain scission of **1e**, forming **1g** and **1h**, is exergonic and proceeds without a significant reaction barrier. Please note the intramolecular rearrangement shown in **1g** since the terminal cation is significantly stabilized by the neighbored F atoms. **1g** reacts with water, forming **1k** or **1l** by a barrier-free reaction, while **1h** is stable in aqueous solution. A direct reaction of **1e** with water to **1o** and **1h** is exergonic. However, the reaction barrier is 89.6 kJ/mol and, thus, too high for a reaction at room temperature. This is also the case for the reaction to **1j** and **1n**. Since the chain scission of **1e** to **1g** and **1h** is exergonic and 30 kJ/mol lower than the chain scission of **1e** to **1i** and **1j**, the latter endergonic reaction should play a minor role. Thus, **1i**, **1j**, and **1m** are probably not formed. In addition, a bond scission at **1e**, forming a H or a F radical, is unlikely since both reactions are strongly endergonic. Thus, the formation of **1h** and **1l** is most important for RIGI since all other reaction pathways are either endergonic or possess a significant reaction barrier. However, Scheme 2 assumes that ionized PVDF relaxes before any reaction takes place, since static quantum chemistry calculations are limited to stationary points of the potential energy surface. Thus, the role of the first few picoseconds after PVDF ionization are barely considered, which might contribute significantly to a release of HF. In addition, cross-linking was not investigated as it has been extensively examined in other studies [72].

**Figure 5:**
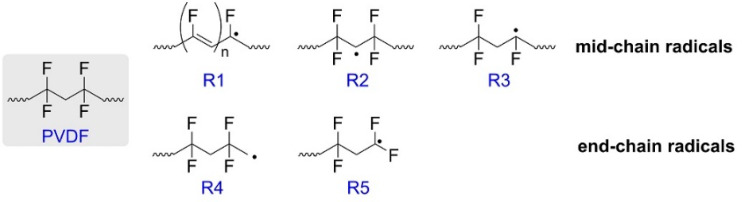
Observed radiation-induced PVDF radicals [76].

The radiation chemistry of pristine PVDF was experimentally studied intensively using mostly γ-radiation [72,73,74]. According to EPR studies [75,76], there are mainly five types of PVDF radicals formed during irradiation (Figure 5), consisting of one olefinic radical due to dehydrofluorination (**R1**), two mid-chain aliphatic radicals (**R2**, **R3**), as well as two end-chain aliphatic radicals (**R4**, **R5**). Thus, experimentally observed radicals formed by neat PVDF are similar to our DFT calculations, which study the reaction in an aqueous environment. Polyene radicals **R1** might be formed within the polymer bulk material as a result of local heating due to relaxation processes of the ionized species in the first few picoseconds after ionization. We have limited the considered structures to mono-alkenes (e.g., **1l**) since we are interested mainly in processes at the polymer surface. Dehydrofluorination of PVDF thin films during irradiation was studied by Makuuchi et al. determining a radiation chemical yield of G(HF) = 1–4 per 100 eV, however, strongly depending on the HF extraction strategy due to follow-up reactions between HF and PVDF radicals [77,78].

**Figure 6:**
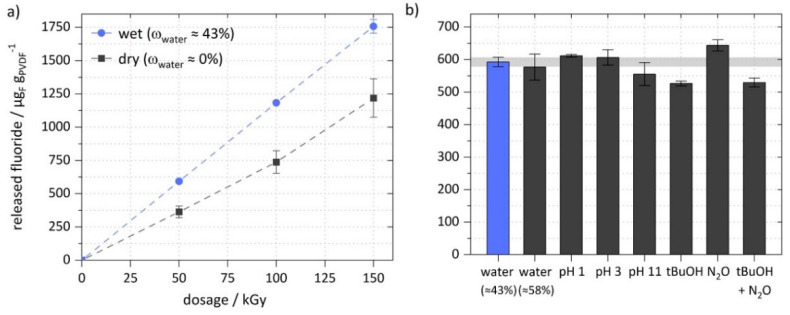
Amount of fluoride released after EB irradiation of PVDF, determined by ion chromatography (*n* = 3). (**a**) Influence of water and irradiation dosage on extracted fluoride and (**b**) effects of scavenging individual water radiolysis products at an irradiation dosage of 50 kGy.

**Scheme 3:**
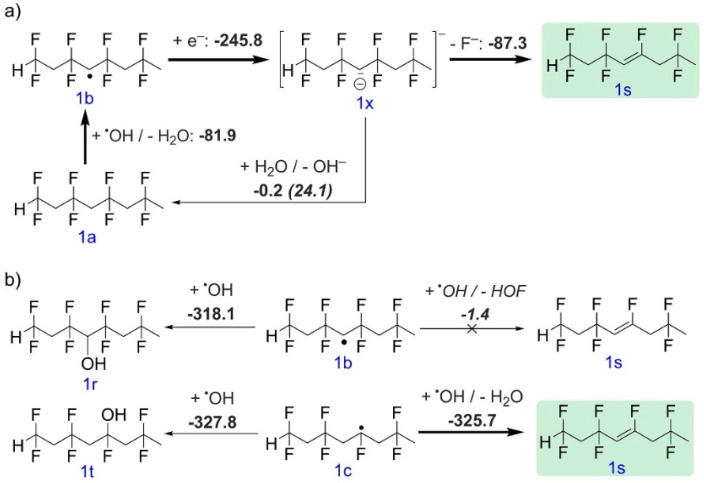
Follow-up reactions of water radiolysis products with PVDF, inhibiting RIGI or forming alkene groups, most importantly **1s**. (**a**) Possible pathway for defluorination according to Figure 6 and (**b**) radical recombination reactions competing with alkene-forming reactions induced by OH radicals. Further reactions are given in Scheme S1. Gibbs free energies are given in kJ/mol. Please note, the reaction from **1b** to **1s** is exergonic, but a direct reaction pathway could not be found.

Thus, dehydrofluorination is an essential part of PVDF radiation chemistry, and since it is easily accessible experimentally, irradiation studies were performed to determine the HF amount released depending on various parameters (Figure 6). By extraction with an alkaline buffer, the amount of fluoride was confirmed independently by ion chromatography and UV–VIS photometry (Table S1 in Supplementary Materials). The amount of fluoride released showed a positive correlation toward the irradiation dosage, for both dry and wetted membranes, i.e., pristine and impregnated PVDF, respectively (mass fraction of water ω ≈ 43%, Figure 6a). The measured absolute quantity was always less in the case of dry vs. impregnated membranes, e.g., 1219 vs. 1757 µg/g_PVDF_ at a dose of 150 kGy, respectively. This fact is also reflected in the G values per 100 eV related to the irradiated PVDF mass: G_dry_(HF) = 3.86 ± 0.24 vs. G_wet_(HF) = 6.00 ± 0.04, respectively. The difference of 2.14 per 100 eV is, thus, the result of synergetic effects induced by water. G_wet_ is higher than the literature values (1–4 per 100 eV) [78], which can be explained by the much larger inner surface area of the porous membrane compared to thin films and a facilitated release of HF due to water solvation effects. To gain further insights into the mode of action, irradiation studies in various aqueous systems were performed at 50 kGy (Figure 6b). Interestingly, by increasing the water amount to ω ≈ 58%, no significant changes in amount of fluoride release were observed. This could indicate that a saturation effect takes place when the whole surficial interface is covered by water molecules. By using perchloric acid to produce acidic solutions at pH 1 (H^+^ + **‧**H → **‧**H_2_^+^) and pH 3 (H^+^ + e^−^_aq_ → **‧**H), effects of H radicals and solvated electrons could be investigated [79]. Despite possible reactions (e.g., **1a**→**1c**, Scheme 1) no significant effects could be observed, leading to the conclusion that neither H radicals nor solvated electrons may be the major cause for dehydrofluorination. However, by scavenging OH radicals with alkaline solutions and, especially, with tBuOH [79], a reduction from 592.5 to 526.4 µg/g_PVDF_ could be detected. Furthermore, by transferring solvated electrons into OH radicals using nitrous oxide, an inversed effect was found (N_2_O + e^−^_aq_ → N_2_ + OH^−^ + **‧**OH) [79]. This indicates that OH radicals could increase the release of fluoride/HF. Such a reaction pathway could be the initial H abstraction from PVDF **1a** by a OH radical-forming **1b** and the immediate capture of a solvated electron forming **1x**, which is able to release F^−^ in order to react to the alkene **1s** (Scheme 3a). Since using N_2_O increased the amount of released fluoride, we assume that N_2_O was not able to scavenge all solvated electrons, especially those that are distributed within the PVDF polymer chains. However, the major cause of dehydrofluorination seems to be, indeed, the direct ionization of PVDF by electron beam (Figure 6a, dry samples). Please note, these effects are not considered in the quantum chemistry calculations, since initial relaxation processes of the formed excited cations to the ground state cannot be described by the employed approach. Dehydrofluorination is specific to any radiation-induced processing of PVDF, not just RIGI. However, by applying an aqueous environment during irradiation, HF can be dissolved for further processing and, thus, facilitates safer handling, which is an advantage over the otherwise occurring emission of HF gas. Since RIGI should be mostly universal toward common polymer materials, the use of different polymer types could mitigate the HF issue.

#### 3.1.3. Potential Inhibition of RIGI by Water Radiolysis Products

We identified **1b**, **1c**, and **1h** as overall stable radicals formed by our molecular model **1a** of PVDF. OH and H radicals, as well as solvated electrons, are expected to possess high mobility in aqueous solution and, thus, might interact with PVDF to inhibit the immobilization process. In the case of the OH radical, we found that a radical recombination reaction forming a covalent bond is strongly exergonic and proceeds without any significant reaction barrier (Scheme 3b). Interestingly, the abstraction of a H atom neighbored to the polymer radical center in **1c** leading to **1s** is strongly exergonic, too. The reaction of **1b** to **1s** is not possible since an O-F bond is overall unstable, and a direct reaction pathway could not be identified based on the screened reaction coordinate. In the case of the H radical, the formation of a double bond by atom abstraction and single-bond formation by radical recombination are possible (Scheme S1). Since all these reactions proceed without a significant reaction barrier, it seems reasonable that alkene groups can be formed during RIGI. In contrast to the single bonds formed by radical recombination or the reaction of the solvated electron with radicals formed by PVDF, these double bonds facilitate the immobilization of radicals formed by the solute (see Section 3.3).

### 3.2. Reactivity of Glycine and Taurine

#### 3.2.1. Reactivity of Glycine

**Scheme 4:**
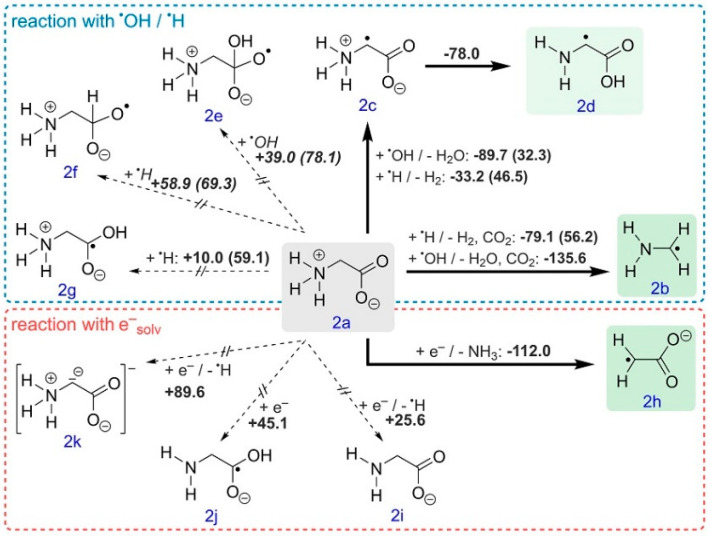
Reactions of glycine with water radiolysis products. Most important for RIGI is **2b** by OH radicals, **2h** by solvated electrons, and, to a lesser extent, **2d** by H radicals. Values in parentheses are activation barriers. All values include thermodynamic corrections and are given in kJ/mol.

We found that the hydroxyl radical most likely abstracts a hydrogen atom at the amine group of glycine **2a**, releasing CO_2_ and forming **2b** since this process occurs without a significant reaction barrier (Scheme 4). Hydrogen atom abstraction by ·OH at Cα, forming **2c**, and subsequent tautomerization are also possible, but the initial step possesses an activation energy of about 32.3 kJ/mol. In contrast, H radicals most likely react with **2d** instead of **2b** since this reaction possesses the lowest reaction barrier. Additionally, ·H can also add to the carboxyl group, forming **2g**. Reactions of **2a** with ·OH to **2e** or with ·H to **2f** can be excluded since both reactions are endergonic. Most likely is the formation of **2h** by releasing ammonia for the solvated electron. Unfortunately, our approach does not allow determining the reaction barrier. However, a first-principles molecular dynamics (FPMD) study revealed this mode of action as well [80]. Initially, the electron binds to the carboxyl group of glycine. Subsequently, ammonia is released as soon as the p-orbital at the carbon atom of the carboxyl group and the Cα-N bond are parallelly oriented. We observed a similar behavior in our tight-binding MD simulations. Experimental studies reported a deamination of glycine by solvated electrons, too [81]. Furthermore, the deamination is inhibited in ß-alanine, which indicates that the position of the carboxyl group plays an essential role in the deamination of amino acids and, thus, supports the computational results [81]. In addition to **2h**, we found the formation of **2j** in our MD simulations. However, this reaction is endergonic, and thus, **2j** can be only an intermediate. The formation of **2j** in our tight-binding simulations is enhanced by the poor description of the solvated electron favoring the addition to glycine. Other reaction products were not observed in our MD simulations. **2i** by releasing ·H is obtained in the static quantum chemistry calculations if reduced **2a** is optimized. This reaction is as unlikely as releasing ·H from Cα, forming **2k**. Thus, **2b** by ·OH, **2d** by ·H, and **2h** by e^−^ can be formed and should facilitate the immobilization of these compounds on PVDF. A direct ionization of glycine and subsequent reactions might result in compounds suitable for immobilization, too. However, the proportion of glycine in the system is overall small, and thus, direct ionization and follow-up transformations were not investigated. Most important for immobilization should be reactants formed by radiolysis of water.

#### 3.2.2. Reactivity of Taurine

**Scheme 5:**
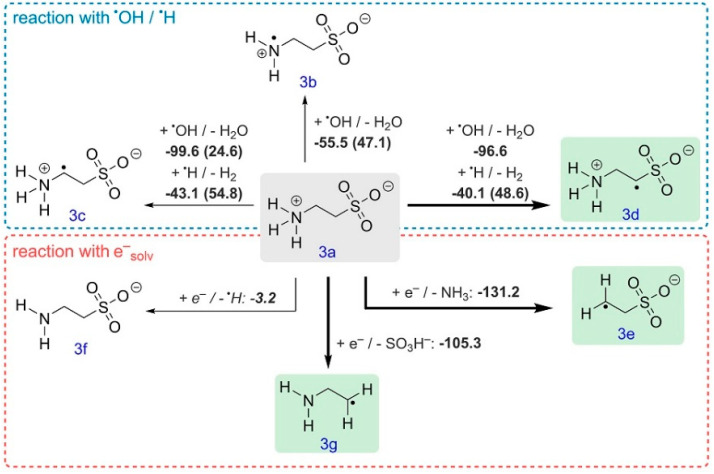
Reactions of taurine with water radiolysis products. Most important for RIGI is **3d** by OH radicals and **3e** as well as **3g** by solvated electrons. Values in parentheses are activation barriers. All values include thermodynamic corrections and are given in kJ/mol.

Reactivity of taurine differs strongly to glycine since more than one aliphatic group separates both functional groups (Scheme 5). The abstraction of a H atom at the amine group by ·OH is unlikely since this pathway possesses an activation barrier of 47.1 kJ/mol. For ·H, a direct reaction pathway from **3a** to **3b** could be even excluded. The release of CO_2_ plays a crucial role in the H atom abstraction at the amine group in glycine. Since this is not possible in taurine, the H atom abstraction by ·H and ·OH at the carbon atom neighbored to the sulfonic acid group is most likely. The reaction from **3a** to **3d** proceeds even without a significant activation energy for ·OH. We found two potential exergonic reactions for the solvated electron. First, ammonia might be released, forming **3e**. Second, sulfurous acid might be released, forming **3g**. The formation of **3f** by release of ·H seems unlikely. In 90 of 100 MD simulation runs, the C-S bond is strongly elongated or even broken at the end of the 20 ps run, while ammonia is released in the remaining simulations. Unfortunately, neither our static quantum chemistry calculations nor the tight-binding simulations are capable to determine the transition state from **3a** to **3e** or **3g** due to the poor description of the solvated electron. Thus, we cannot conclude whether the formation of **3e** or **3g** is kinetically inhibited or which product is preferred. Therefore, **3d**, **3e**, and **3g** are considered reactants.

### 3.3. Grafting

**Scheme 6:**
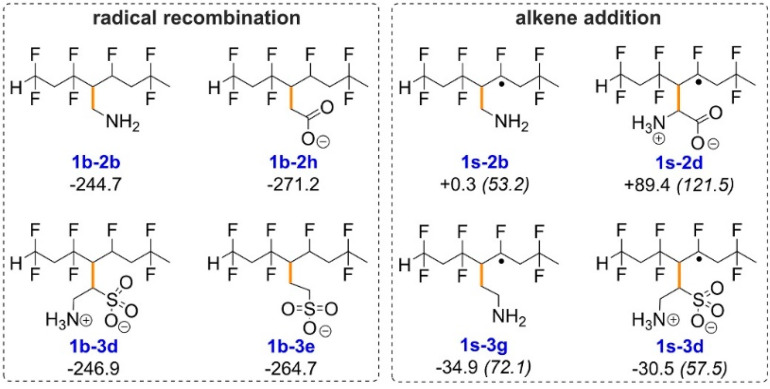
Most likely grafting structures for RIGI and PVDF due to radical recombination and alkene addition reactions, respectively, corresponding to the possible transformation products identified as most important in our calculations. The orange color highlights the formed covalent bond. Further reactions are given in Scheme S2 and Scheme S3. The free reaction enthalpy is given in kJ/mol and refers to the reaction a + b → a − b. For example, a is reactant **1b** and b is reactant **2b** for the product **1b**-**2b**. Values for found transition states are given in parentheses.

Two general reaction pathways are possible for immobilization by a covalent bond. First, two radicals can recombine. These reactions are strongly exergonic and proceed without a significant activation energy (see the most likely structures in Scheme 6 and Scheme S2). Indications for a significant reaction barrier were not observed in a CASSCF(2,2) calculation as well (Figure S1. Thus, radicals can recombine as soon as they approach each other. Second, the radical can add to a formed alkene group (see Scheme S3) for which the following general behavior was observed: Radicals that are not stabilized by π-orbitals or lone pairs at neighbored atoms have an exergonic reaction enthalpy (see **1s**-**3d** and **1s**-**3g** in Scheme 6). Neighbored π-orbitals or lone pairs have two effects: the stability of the product and the reaction barrier decrease (compare **1s**-**2b** and **1s**-**3g** in Scheme 6). Forming a covalent bond is even strongly endergonic, where the radical is stabilized by a conjugated π-system, e.g., for **2d**. Independent of the radical, the reaction barrier is at least 50 kJ/mol, and thus, these reactions should proceed much slower than the radical recombination reactions. The high reaction barrier can be attributed to missing attractive covalent interactions that pre-arranges both reactants before bond formation. Thus, thermodynamic corrections (rotation, translation, vibration) and zero-point energy corrections result in an overall high energy barrier.

Successful immobilization of glycine (Table 1) and taurine (Table 2) could be confirmed by X-ray photoelectron spectroscopy. Due to its high surface sensitivity, XPS is one of the few methods for verifying the mono-molecular grafting of small non-vinyl substances. The pristine PVDF membrane as well as the 0 kGy reference samples did not show any detectable N content. In comparison, the PVDF-*graft*-glycine samples irradiated at 150 kGy showed an increase in N content (N/C = 0.20–0.49%), giving strong evidence for covalent immobilization of the amino acid. Interestingly, higher concentrations (ω = 1% vs. ω = 0.1%) resulted in actual lower amounts of immobilized glycine (N/C = 0.20% vs. 0.49%, respectively). This could be explained by an increase in competitive reactions, e.g., with each other or with water radiolysis products, especially since radical sites on such small molecules are easily accessible. Irradiation dosage seems to have a significant effect on the grafting yield, as can be seen for the 50 kGy samples having always lower (N/C = 0.18%) or even no measurable nitrogen content. It should be noted that the O content varies greatly, especially due to reactions with water radiolysis products and oxidation processes, which makes it rather unsuitable for the verification of grafting. In contrast to the non-polymerizable glycine used in the RIGI approach, the classical RIGP approach has to use more reactive compounds such as 2-aminoethyl methacrylate (AEMA, Table 1). Interestingly, the N content was lower for PVDF-*g*-AEMA (N/C = 0.16–0.48%) than for PVDF-*g*-glycine (N/C = 0.49%, ω = 0.1%). However, while in RIGI, a higher solute concentration will reduce grafting yield, in RIGP, a higher monomer concentration will increase the yield [33].

Similar trends in immobilization were observed when using taurine. Again, detectable amounts of N were only found in the case of irradiated samples, supporting the strong evidence for a covalent coupling mechanism. In addition, the attachment of taurine with its sulfate group could be proven by an increase of the S content from 0% (pristine PVDF and 0 kGy reference) to a maximum of S/C = 0.22% (ω = 0.1%, 150 kGy). The same sample also possessed the highest N content, with N/C = 0.57%. It appears that an impregnation solution with a mass fraction of ω = 0.1% (corresponding to 13 mM glycine or 8 mM taurine) could be the sweet spot for RIGI when using small molecules. The 1% and 0.01% solutions led to less or even no grafting, respectively. It should be noted that according to Scheme 4 and Scheme 5, deamination could be a potential reaction pathway, which is why the measured amounts of N could underestimate the actual immobilized molecules. Likewise, the release of SO_3_H^−^ is possible, which could explain differences in N and S content.

**Figure 7:**
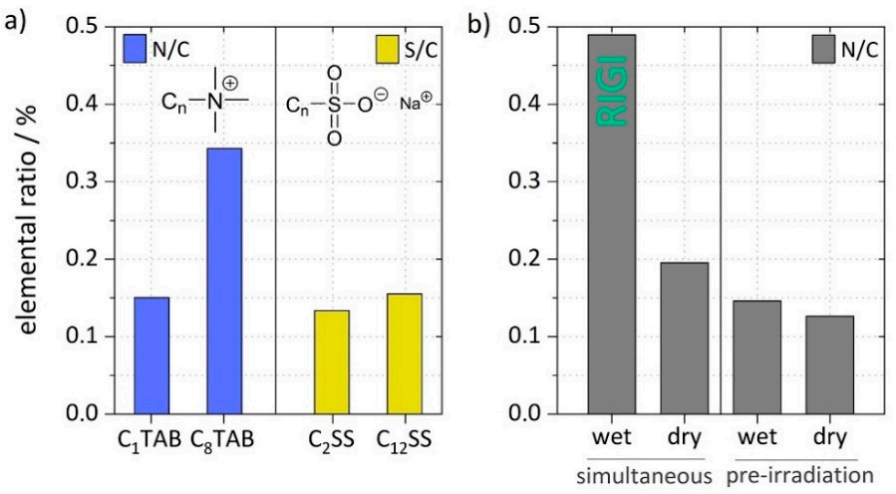
Influence of hydrophobicity, charge, water content, and irradiation method: (**a**) immobilization of a cationic and an anionic surfactant each with two different alkyl chain lengths (10 mM, 150 kGy) in order to investigate the influence of hydrophobicity and charge and (**b**) effect of water content and irradiation method on N/C grafting yield (glycine, ω = 0.1%, 150 kGy). XPS data can be found in Table S2.

Immobilization of soluble surfactants allowed a preliminary investigation of the influence of hydrophobicity (length of alkyl chains) and charge (cationic alkyl trimethylammonium bromide (C_n_TAB) and anionic alkyl sodium sulfate (C_n_SS)). The N/C and S/C elemental ratios indicate that a general affinity to the trunk polymer could facilitate coupling, since radical recombination reactions could take place preferentially (Figure 7a). The used PVDF membrane is a hydrophobic and slightly negative charged polymer due to OH^−^ adsorption from water [82]. Thus, a longer alkyl chain could increase the hydrophobic interactions and facilitate the radical recombination responsible for RIGI (N/C = 0.15% for C_1_TAB vs. N/C = 0.34% for C_8_TAB). In the case of the anionic surfactant, however, this effect is hardly noticeable (S/C = 0.13% for C_2_SS vs. S/C = 0.16% for C_12_SS), which could be explained by electrostatic interaction. In the case of the cationic surfactant, the electrostatic attraction and, thus, the probability of successful grafting are increased. Please note that the formation of stable anionic species by capturing a solvated electron could also reduce the grafting yield. This will be investigated in future work. As assumed in the RIGI mechanism, grafting is achieved by radical reactions between polymer and solute radicals, with the latter, in particular, being generated by radiolysis products of water. Consequently, the presence of water as well as the irradiation method should play a decisive role for the yield (Figure 7b; glycine, ω = 0.1%, 150 kGy). The RIGI approach, as proposed in this work, is based on the simultaneous EB irradiation of an aqueous impregnated membrane, resulting in N/C = 0.49%. Drying of the sample at 50 °C overnight leads to a dramatic reduction in the N content (N/C = 0.20%), which could be explained by a reduced solute radical yield. However, unlike in the case of the 0 kGy reference, immobilization is still happening, possibly due to residual water, moisture from the atmosphere, or the formation of reactive species due to irradiation itself. Thus, grafting was also observed in the pre-irradiation method but with even further reduced yields (N/C = 0.13–0.15%). It is known that PVDF can form stable radicals (up to minutes) [76], especially in the crystalline regions. This might possibly be the reason for the present but marginal coupling.

## 4. Conclusions

In this study, we proposed a new terminology for an advanced type of grafting reaction: radiation-induced graft immobilization (RIGI). In contrast to the established radiation-induced graft polymerization (RIGP) approach, RIGI can use non-vinyl compounds such as small organic molecules (e.g., glycine and taurine), other organic polymers, and even biomacromolecules such as enzymes. Thus, high versatility is achieved, and environmentally harmful or potentially toxic vinyl substances (e.g., acrylates or styrenics) are avoided. The reaction mechanism for functionalization of PVDF polymer membranes was investigated by experimental and computational approaches. By using electron beams (EBs) as a form of high-energy ionizing radiation, the formation of covalent bonds is induced, primarily by radical recombination reactions and to a lesser extent by alkene addition reactions. By using water as a solvent for the modifying agent, the in-situ-generated water radiolysis products (e.g., OH and H radicals or solvated electrons) can transfer the solute into reactive radical species and enrich radical sites on the polymer surface. Dehydrofluorination occurs mainly as a consequence of direct PVDF ionization and strongly depends on the applied dosage. This is supported by the computational studies on the ground-state reactivities since a direct reaction pathway for release of fluoride by OH radicals or solvated electrons could not be found. The release of HF is specific to any radiation-based processing of fluoropolymers such as PVDF. Nevertheless, the RIGI approach can compensate for this drawback by performing irradiations in an aqueous environment, which facilitates HF extraction and further cleaning. The grafting yield for the supposed mono-molecular immobilization of glycine and taurine was determined by XPS. The highest N/C elemental ratio was 0.49% for glycine and 0.57% for taurine. In both cases, the optimal conditions were an irradiation dosage of 150 kGy and an impregnation solution with a mass fraction of ω = 0.1%. However, the conditions might be only suitable for small molecules with high mobility like the two model molecules investigated. Furthermore, hydrophobic chains attached to cations seem to enhance covalent attachment. This study confirmed previously described experimental results for the first time by elucidating the underlying reaction mechanism. This knowledge could be used to facilitate the adjustment of reaction parameters and to promote an expansion of the RIGI process to the immobilization of molecular catalysts or functional biomolecules. Thus, RIGI provides a unique platform technology to generate functional polymer membranes by a roll-to-roll process.

## Figures and Tables

**Table 1:** XPS investigations of pristine PVDF membrane and PVDF modified with glycine (mass fractions ω = 0.1–1% and dosages of 0, 50, and 150 kGy), as well as AEMA immobilization (RIGP mechanism) in comparison.

Sample	Elemental Composition/at%					Elemental Ratio/%
	C	F	O	N	Si *	N/C
PVDF, pristine	52.69	44.90	2.14	0.00	0.28	0.00
PVDF-*g*-glycine, 0.1%, 0 kGy	53.34	43.94	2.33	0.00	0.40	0.00
PVDF-*g*-glycine, 0.1%, 50 kGy	49.29	49.07	1.37	0.09	0.20	0.18
PVDF-*g*-glycine, 0.1%, 150 kGy	49.00	49.45	1.19	0.24	0.13	0.49
PVDF-*g*-glycine, 1%, 0 kGy	51.58	46.76	1.46	0.00	0.20	0.00
PVDF-*g*-glycine, 1%, 50 kGy	56.36	38.27	4.70	0.00	0.67	0.00
PVDF-*g*-glycine, 1%, 150 kGy	50.45	47.69	1.56	0.10	0.19	0.20
PVDF-*g*-AEMA, 0.2%, 150 kGy	55.02	42.25	2.30	0.09	0.34	0.16
PVDF-*g*-AEMA, 2%, 150 kGy	52.62	44.43	2.50	0.25	0.20	0.48

* Si appears to be an impurity from the pristine PVDF membrane, e.g., from the manufacturing process.

**Table 2:** XPS investigations of pristine PVDF membrane and PVDF modified with taurine (mass fractions ω = 0.1–1% and dosages of 0, 50, and 150 kGy).

Sample	Elemental Composition/at%						Elemental Ratio/%	
	C	F	O	N	S	Si *	N/C	S/C
PVDF, pristine	52.69	44.90	2.14	0.00	0.00	0.28	0.00	0.00
PVDF-*g*-taurine, 0.1%, 0 kGy	50.97	47.31	1.46	0.00	0.00	0.26	0.00	0.00
PVDF-*g*-taurine, 0.1%, 50 kGy	54.86	41.35	3.27	0.10	0.05	0.37	0.18	0.09
PVDF-*g*-taurine, 0.1%, 150 kGy	50.59	46.20	2.57	0.29	0.11	0.24	0.57	0.22
PVDF-*g*-taurine, 1%, 0 kGy	45.16	53.91	0.78	0.00	0.00	0.14	0.00	0.00
PVDF-*g*-taurine, 1%, 50 kGy	51.41	47.32	1.07	0.00	0.00	0.20	0.00	0.00
PVDF-*g*-taurine, 1%, 150 kGy	45.63	53.02	1.04	0.10	0.06	0.14	0.22	0.13

* Si appears to be an impurity from the pristine PVDF membrane, e.g., from the manufacturing process.

## Data Availability

Input and output files of the static quantum chemistry calculations were uploaded to the NOMAD repository, available by PID HpfuIDx9Sfqfza0CFEOmFQ.

## Supplementary Materials

The following are available online at https://www.mdpi.com/article/10.3390/polym13111849/s1, Figure S1: Comparison of calculated potential energy surface for **1c**-**2d** formed by **1c** and **2d**, Scheme S1: Reactions with water radiolysis products inhibiting RIGI on PVDF, or forming alkene groups, Scheme S2: Investigated radical recombination reactions, Scheme S3: Investigated alkene addition reactions, Table S1: Investigation of PVDF dehydrofluorination depending on irradiation dosage and water content, Table S2: XPS investigations of surfactant and glycine immobilization.
