# Supplementary material for: Radiation-Induced Graft Immobilization (RIGI): Covalent Binding of Non-Vinyl Compounds on Polymer Membranes
Source: Polymers (Basel). 2021 Jun 2;13(11):1849. doi: 10.3390/polym13111849 (PMC8199689; doi:10.3390/polym13111849)
Supplement: Supplementary file 1 [file polymers-13-01849-s001.zip › polymers-1213438-supplementary.pdf]

polymers

MDPI

Supplementary Materials

Radiation-Induced Graft Immobilization (RIGI): Covalent Binding of Non-Vinyl Compounds on Polymer Membranes

Martin Schmidt [1], Stefan Zahn [1], Florian Gehlhaar [1], Andrea Prager [1], Jan Griebel [1], Axel Kahnt [1], Wolfgang Knolle [1], Robert Konieczny [1], Roger Gläser [2] and Agnes Schulze [1,*]

[1] Leibniz Institute of Surface Engineering (IOM), Permoserstr. 15, 04318 Leipzig, Germany;
[2] Institute of Chemical Technology, Leipzig University, Linnéstraße 3, 04103 Leipzig, Germany;
* Correspondence: agnes.schulze@iom-leipzig.de

Keywords: polymer surface; membrane; radiation-induced grafting; electron beam; reaction mechanism; DFT; MD

Table S1. Investigation of PVDF dehydrofluorination depending on irradiation dosage and water content.

Given are the amount of extracted fluoride in $\mu g/g_{PVDF}$ and the (relative) standard deviation, (R)SD. Two different methods, ion chromatography (n = 3), and Spectroquant® UV/Vis assay (n = 5), respectively, were used.

Sample		Ion Chromatography (HPIC)			Spectroquant® Assay (UV/Vis)		
		$\mu g_F/g_{PVDF}$	SD	RSD / %	$\mu g_F/g_{PVDF}$	SD	RSD / %
dry ($\omega_{water} \approx 0\%$)	0 kGy	0.0	0.0	–	4.5	7.2	–
	50 kGy	363.1	43.3	11.9	353.4	9.3	2.6
	100 kGy	737.9	85.1	11.5	757.2	26.3	3.5
	150 kGy	1219.4	143.9	11.8	1229.5	82.3	6.7
wet ($\omega_{water} \approx 43\%$)	0 kGy	0.0	0.0	–	-6.9	30.2	–
	50 kGy	592.5	14.3	2.4	584.6	38.9	6.7
	100 kGy	1183.5	10.8	0.9	1082.9	50.5	4.7
	150 kGy	1757.2	51.1	2.9	1687.3	40.8	2.4

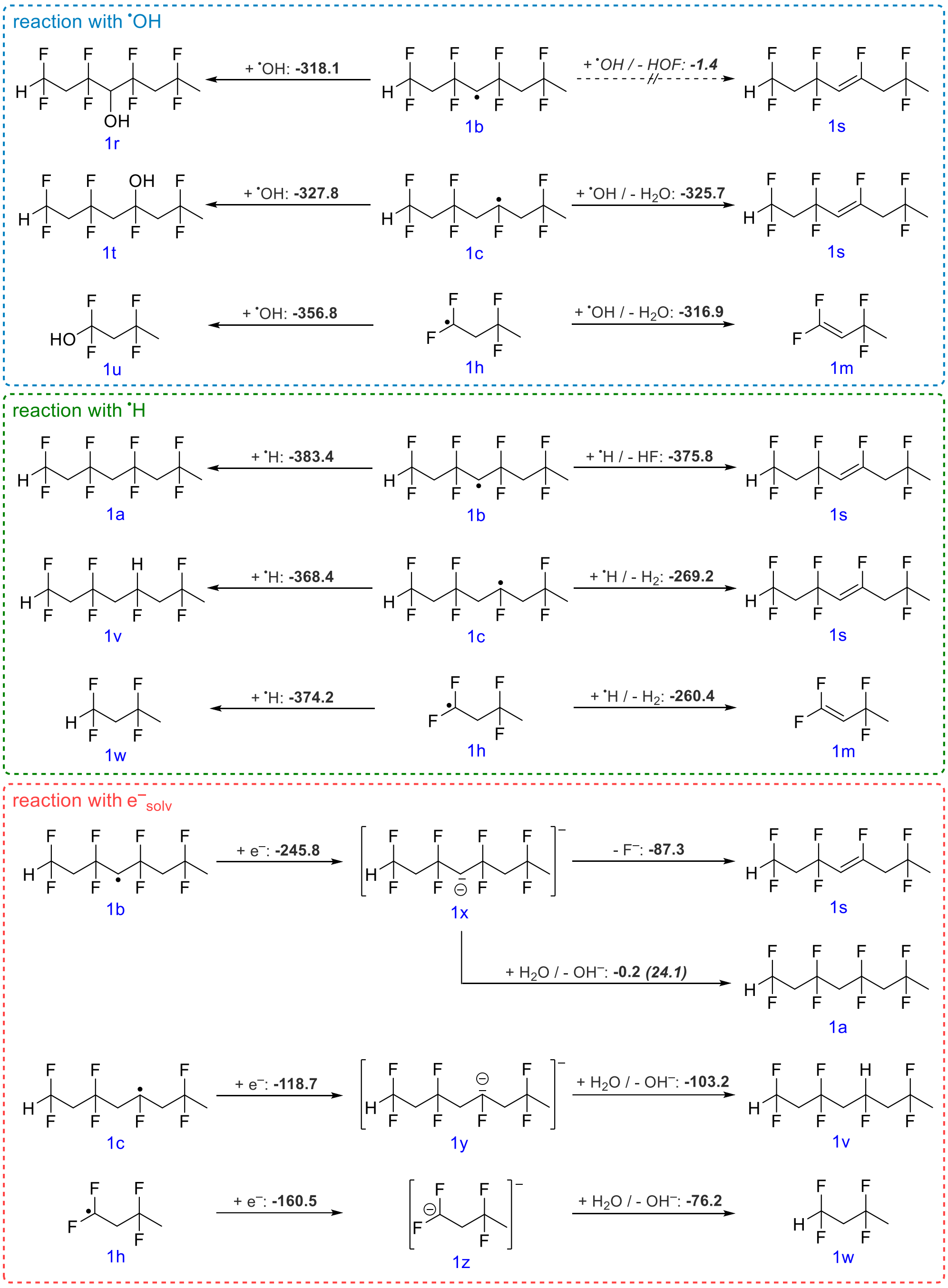

Scheme S1. Reactions with water radiolysis products inhibiting RIGI on PVDF, or forming alkene groups.

Free reaction enthalpies are given in kJ/mol. The reaction of **1b** to **1s** is not possible since a direct reaction pathway could not be identified. Interestingly, **1b** reacts with a solvated electron to **1x** and finally to **1s** by releasing a fluoride anion. A proton transfer from water to **1x** forming **1a** and a hydroxide anion is unlikely because this reaction possesses a barrier of about 24.1 kJ/mol and is less exergonic (-0.2 kJ/mol). Please note, the equilibrium between **1x** and **1a** will be shifted towards **1a** due to the excess of water. Nonetheless, the reaction from **1x** to **1s** is too strong exergonic and thus, the formation of **1s** should be thermodynamically and kinetically favored.

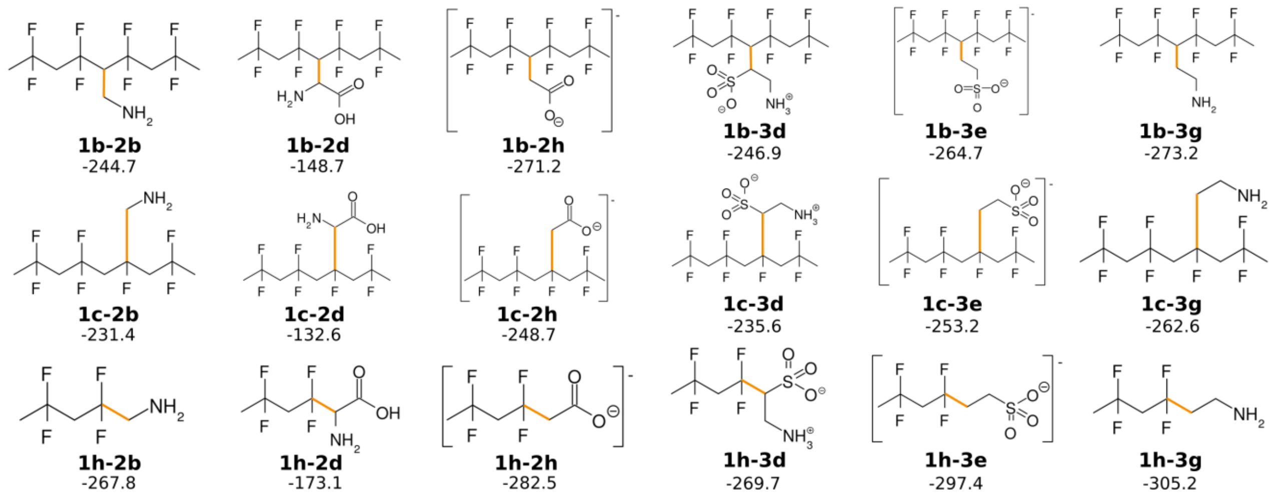

Scheme S2. Investigated radical recombination reactions.

The orange color highlights the formed covalent bond. A transition state could not be found in all cases. The free reaction enthalpy is given in kJ/mol and refers to the reaction a + b → a-b. For example, a is reactant **1b** and b is reactant **2b** for the product **1b-2b**.

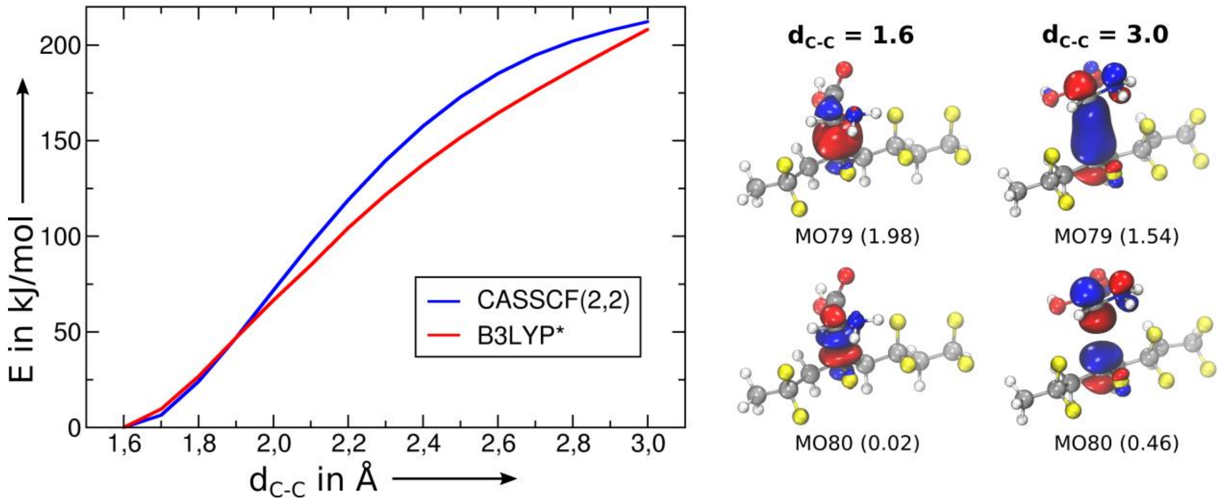

Figure S1. Comparison of calculated potential energy surface for *1c-2d* formed by *1c* and *2d*.

The energy at 1.6 Å distance between both carbon atoms (orange line in Figure S3) was set to 0 kJ/mol. The CASSCF calculation employed the SVP basis set and two electrons in two orbitals. The orbitals of the active space are shown right. The value in bracket is the number of electrons in the respective orbital. The B3LYP* calculation employed a def2-TZVP basis set.

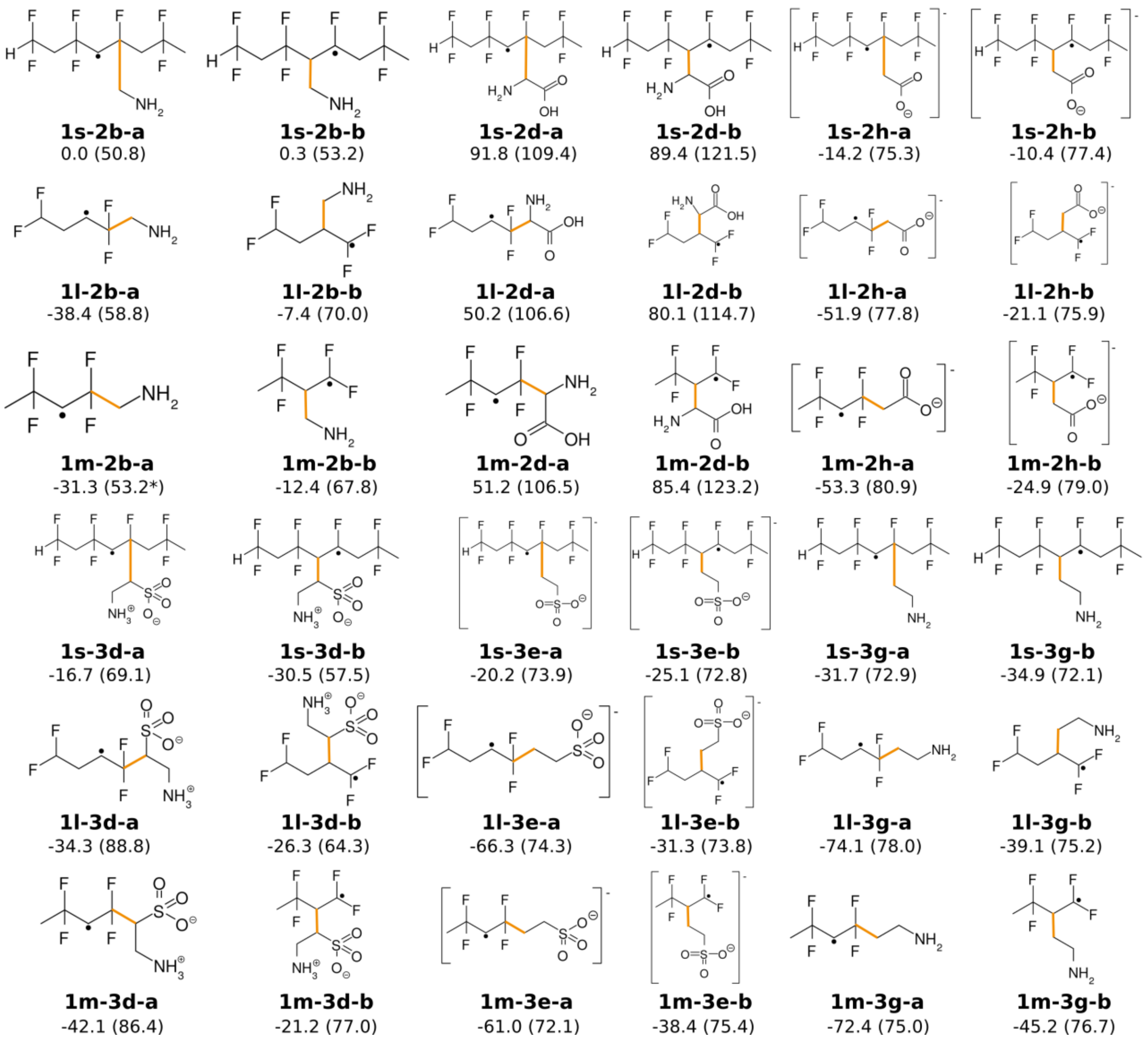

Scheme S3. Investigated alkene addition reactions.

The orange color highlights the formed covalent bond. The free reaction enthalpy is given in kJ/mol and refers to the reaction a + b → a-b. For example, a is reactant **1s** and b is reactant **2b** for the product **1s-2b**. An additional identifier (-a, -b) indicates one of the two possible points of attack where a is at the carbon atom to which an F atom is attached, while b is a carbon atom to which solely H atoms are attached. Transition states (TS) are given in brackets. Please note, a TS for **1m-2b-a** on the DFT energy surface could not be found. However, the entropy during the reaction is significantly decreased compared to the reactants since the number of particles is reduced. Since a precomplex could not be found for the similar reactions indicating no significant attractive interactions before bond formation, we estimated the activation energy of the TS by the difference of the entropy between reactants and product.

Table S2. XPS investigations of surfactant and glycine immobilization.
Cationic C_nTAB (alkyl trimethyl ammonium bromide) and anionic C_nSS (alkyl sodium sulfate) were immobilized using each a 10 mM impregnation solution and an irradiation dosage of 150 kGy. It seems, however, that some impurities were present in case of surfactant immobilization. Glycine was coupled using 150 kGy, impregnation solutions of $\omega = 0.1\%$, and different irradiation strategies (sim = simultaneous irradiation of impregnated membranes; pre = pre-irradiation method, *i.e.*, previous irradiation of the membrane, and subsequently, coupling step *via* impregnation).

Sample	Elemental Composition / at%						Elemental Ratio / %	
	C	F	O	N	S	Si	N/C	S/C
PVDF, pristine	52.69	44.90	2.14	0.00	0.00	0.28	0.00	0.00
C_1TAB, 10 mM, 150 kGy	53.24	43.87	2.36	0.08	0.10	0.35	0.15	–
C_8TAB, 10 mM, 150 kGy	52.52	44.80	2.19	0.18	0.13	0.19	0.34	–
C_2SS, 10 mM, 150 kGy	52.31	45.54	1.79	0.08	0.07	0.22	–	0.13
$C_{12}SS$, 10 mM, 150 kGy	51.57	46.65	1.47	0.05	0.08	0.18	–	0.16
glycine, sim, wet	49.00	49.45	1.19	0.24	–	0.13	0.49	–
glycine, sim, dry	51.16	46.44	2.06	0.10	–	0.24	0.20	–
glycine, pre, wet	54.77	41.28	3.45	0.08	–	0.42	0.15	–
glycine, pre, dry	55.52	39.71	4.14	0.07	–	0.57	0.13	–
